# Gold clusters prevent breast cancer bone metastasis by suppressing tumor-induced osteoclastogenesis

**DOI:** 10.7150/thno.42218

**Published:** 2020-03-04

**Authors:** Zhichao Zhang, Yawen Yao, Qing Yuan, Cao Lu, Xiangchun Zhang, Jinling Yuan, Kaixiao Hou, Chunyu Zhang, Zhongying Du, Xueyun Gao, Xiongsheng Chen

**Affiliations:** 1 Spine Center, Department of Orthopedics, Changzheng Hospital, Second Military Medical University, Shanghai, China.; 2 Department of Chemistry and Chemical Engineering, Beijing University of Technology, Beijing, China.; 3 Center of Excellence for Environmental Safety and Biological Effects, Beijing University of Technology, Beijing, China.; 4 CAS Key Laboratory for the Biological Effects of Nanomaterials and Nanosafety, Institute of High Energy Physics, Chinese Academy of Sciences, Beijing, China.

**Keywords:** Breast cancer bone metastasis, gold clusters, osteoclastogenesis, osteolysis, MDA-MB-231

## Abstract

**Rationale**: Bone is the most frequent site for breast cancer metastasis, which accounts for the leading cause of death in advanced breast cancer patients. Serious skeletal-related events (SREs) caused by bone metastasis have a decisive impact on the life expectancy of breast cancer patients, making breast cancer almost incurable. Metastatic breast cancer cell induced pathological osteoclastogenesis is a key driver of bone metastasis and osteolytic bone lesions. We previously reported that gold clusters can prevent inflammation induced osteoclastogenesis and osteolysis *in vivo*. In this study, we investigated the effects of a BSA-coated gold cluster on metastatic breast cancer-induced osteoclastogenesis *in vitro* and tumor-induced osteolysis *in vivo*, and elucidated its possible mechanism.

**Methods**: Breast cancer cell line MDA-MB-231 was used to evaluate the regulatory effects of gold clusters on breast cancer metastasis and tumor induced osteoclastogenesis *in vitro*. Cell counting kit-8, transwell, wound-healing and colony formation assays were performed to evaluate the effect of gold clusters on proliferation and metastasis of MDA-MB-231 cells. Tartrate-resistant acid phosphatase (TRAP) staining and filamentous-actin rings analysis were used to detect the regulatory effects of gold clusters on MDA-MB-231 cell-conditioned medium (MDA-MB-231 CM) triggered and receptor activator of nuclear factor-κB ligand (RANKL)-induced osteoclastogenesis in mouse bone marrow-derived mononuclear cells (BMMs). A mouse model of breast cancer bone metastasis was used to evaluate the *in vivo* activity of the gold cluster on the tumor induced osteolysis.

**Results**: The gold clusters suppressed the migration, invasion and colony formation of MDA-MB-231 cells in a dose-dependent manner *in vitro*. The gold clusters strongly inhibited both MDA-MB-231 CM triggered and RANKL-induced osteoclast formation from BMMs *in vitro*. Cell studies indicated that the gold clusters suppressed the expression of osteolysis-related factors in MDA-MB-231 cells and inhibited the subsequent activation of NF-κB pathway in BMMs. Treatment with the clusters at a dose of 10 mg Au/kg.bw significantly reduces the breast cancer cell induced osteolysis *in vivo*.

**Conclusion**: Therefore, the gold clusters may offer new therapeutic agents for preventing breast cancer bone metastasis and secondary osteolysis to improve patient outcomes.

## Introduction

Breast cancer is the most prevalent malignant tumor and the leading cause of cancer deaths among females 1. Although local control of the primary site of breast cancer has recently been improved through early diagnosis and appropriate treatment, metastases led to 80% of cancer-associated death 2. Recent advances in cancer treatment have increased patients' life expectancy, but conversely increased the risk of bone metastasis 3. Bone is the most frequent site of metastasis in breast cancer patients, and bone metastasis occur in up to 75% of patients with advanced breast cancer 4,5. Bone metastases secondary to breast cancer can induce severe skeletal-related events (SREs), which will shorten patients' life expectancy to only several years, including pathological fractures, spinal cord compression, humoral hypercalcemia of malignancy (HHM), and pain 6. There is still lacking of ideal solution in preventing breast cancer bone metastasizing. The current method is surgically removing the metastatic lesion in bone and combined with chemotherapy 7. However, it is usually accompanied by a high risk of perioperative complications, and the outcomes are not fully satisfactory 8.

The development of breast cancer bone metastases is a complex process involving crosstalk between bone-seeking cancer cells and bone cells, leading to deregulation of normal bone remodeling processes 9-12. Therefore, it is very important to reduce the metastasis of cancer cells and prevent bone deterioration synchronously in the course of treatment 13. Chemotherapy is the main approach to inhibit the metastasis of cancer cells, but it lacks anti-osteolytic activity 14. Anti-osteoclast bisphosphates that widely used in treating cancer bone metastasis, significantly reduced SRE risk, but did not improve survival in breast cancer patients with bone metastases (BCBM) 15. Furthermore, not all breast cancer patients respond to bisphosphonates, and severe toxicities preclude the use of bisphosphonates, such as renal impairment and osteonecrosis of the jaw 16. Therefore, to suppress the breast cancer bone metastasis and the tumor cell induced osteolysis, current treatments mainly use combination therapy 13. It could be beneficial to develop a reagent that simultaneously suppresses tumor induced osteoclastogenesis and cancer cell invasion to treat breast cancer bone metastasis.

In recent years, gold nanomaterials have attracted wide attention in various biomedical applications due to their biocompatibility, easy synthesis, characterization and surface modification, as well as their unique physicochemical properties 17-19. In the theranostics of breast cancer metastasis, previous research of gold nanomaterials only focused on drug delivery, imaging or adjuvant therapy 20-22. However, the intrinsic therapeutic activity of gold nanomaterials on breast cancer bone metastasis remains unclear to date. Gold clusters prepared by biomolecules are novel gold nanomaterials with ultrasmall size, good biocompatibility and intrinsic biomedical activity, thus exhibited great potentials in biomedical applications 18,19. We have found that peptide-coated gold clusters possessed osteoclastogenesis inhibitory activity and prevented inflammation induced bone destruction effectively *in vivo* 23,24. Moreover, such gold clusters revealed potential anti-tumor activities in certain cancer cells, such as cervix carcinoma (Hela), chronic lymphocytic leukemia (MEC-1), non-small cell lung carcinoma (A549), nasopharyngeal cancer (CNE1), and malignant glioblastoma (U87-MG) 25-29. Considering the key role of osteoclast and tumor cell activities in bone metastasis of breast cancer, we speculate the gold clusters may provide an exciting strategy to treat breast cancer bone metastasis.

Albumin is the most abundant serum protein that widely used in applications of nano-pharmaceutics for its excellent biocompatibility 30-32. Albumin-bound paclitaxel (Abraxane) has been the first nanotechnology-based drug on the market, indicating that protein can facilitate the clinical transformation of nanomedicine 30. In this study, we use the bovine serum albumin (BSA) to synthesize a gold cluster, which has been proved to be biocompatible 33-36. Our results indicated the gold cluster effectively inhibited the migration, invasion and colony formation of human breast cancer cells MDA-MB-231 and tumor-induced osteoclastogenesis *in vitro*. The gold cluster also suppressed the RANKL-induced differentiation of osteoclasts from murine bone marrow mononuclear cells (BMMs) *in vitro*. In a mouse model of breast cancer bone metastasis, treating with the gold cluster markedly ameliorate the tumor-induced osteolysis *in vivo*. These data proved the gold clusters can effectively suppress metastatic breast cancer-induced osteoclastogenesis and osteolysis *in vivo*, and with activity in preventing the migration, invasion and colony formation of breast cancer cells. Based on these results, gold cluster may provide a promising therapeutic strategy for treating breast cancer bone metastasis.

## Methods

### Preparation and characterization of BSA-Au clusters

The BSA-coated gold clusters were prepared as we previously reported 33,34. In brief, 2 mL of freshly prepared HAuCl_4_ aqueous solutions (4.375 mM) was added into equal volume of BSA solution (10 mg/mL) under vigorous stirring, then 0.5 mL of 0.5 M NaOH was added, and the final mixture was incubated at dark for 12 h. The product was purified by using 3000G ultrafiltration (Millpore, MWCO: 30 K) to remove free BSA and other ions. The BSA-Au clusters solution was then filtered by a 0.22 μm filtration membrane to eliminate impurities and bacteria. The content of Au in the obtained BSA-Au clusters was evaluated by inductively coupled plasma mass spectrometry (ICP-MS, PerkinElmer). The obtained BSA-Au clusters were then characterized by transmission electron microscope (TEM, JEOL.). And the fluorescence emission and excitation were detected with fluorescence spectrometers (Thermo Fisher Scientific). Nano Measurer 1.2 software was used to analyze the size distribution. The hydrodynamic size of obtained BSA-Au clusters was characterized by dynamic lighting scattering (DLS) (ZEN3700, Malvern, UK). The formula of the clusters was detected by matrix assisted laser desorption/ionization time of flight mass spectrometry (MALDI-TOF-MS) system (ABI) in positive ion mode with α-cyano-4-hydroxycinnamic acid as matrix. To test the stability of BSA-Au clusters in water, saline, DMEM complete medium or α-MEM complete medium respectively, the fluorescence intensity of the clusters was tested by fluorescence spectrophotometer (Thermo Fisher Scientific) over 48 h at room temperature.

### Cell culture

Mouse BMMs cells were harvested from the femur and tibia of 3-4week female C57-6J mice (Beijing Huafukang Bioscience CO. INC), cultured in α-minimum Eagle's medium (α-MEM, Gibco) with 10% of fetal bovine serum (FBS, Gibco), 1% of penicillin-streptomycin solution (Gibco) and 10 ng/μL of mouse macrophage colony-stimulating factor (MCSF, R&D). Human breast cancer cell line MDA-MB-231were purchased from the Cancer Institute and Hospital, Chinese Academy of Medical Science, cultured in Dulbecco's modified Eagle medium (DMEM, Gibco) with 10% of FBS (Gibco) and 1% of penicillin-streptomycin solution (Gibco). All cells were incubated at 37°C, 5% CO_2_ environment and the media were refreshed every 48 h.

### Cellular proliferation

BSA-Au clusters were added to the media at the Au concentration of 10 μM, 20 μM, 50 μM and 100 μM respectively in 96-well plates cultured with 1×10^4^ cells each well. The cell proliferation was assessed by using a cell counting kit (CCK-8, Beyotime Biotechnology Inc). The plates were then incubated for 6 h, 12 h, 24 h and 48 h. At each predetermined time point, the media was replaced with fresh medium containing CCK-8 and incubated for another 2 h. The absorbance was measured at 450 nm with microplate reader (Molecular Devices).

### Mouse BMMs osteoclastic differentiation and tartrate-resistant acid phosphatase (TRAP) staining

Mouse BMMs were extracted by the following procedures: the femurs and tibias were separated after cervical dislocation of the mice. Cut out both ends of the long bone to open up the bone marrow cavity. The bone marrows were washed out from one of the ends by injecting α-MEM medium from the other. The bone marrows were then centrifuged at 1200 rpm for 3 min and resuspended in 5 mL of α-MEM medium. 2 mL of red blood cell lysis buffer (Solarbio) were added to the solution and left alone for 3 min. The solution was centrifuged again at 1200 rpm for 3 min and resuspended in α-MEM medium with 10% FBS. Cells left in the supernatant were collected after 24 h of incubation in 37 ºC, 5% CO_2_ environment. The cells were centrifuged at 1200 rpm for 3 min and resuspended in α-MEM medium with 10% FBS and 20 ng/mL of M-CSF. After another 48 h of incubation in 37 ºC, 5% CO_2_ environment, the cells still in the supernatant were abandoned and the adherent cells were recognized as BMMs. The BMMs cells were cultured in 16-well plate with 1x10^5^ cells each well. We induced osteoclastogenesis by two separate ways, direct method and indirect method. The direct method was to add 50 ng/μL of mouse receptor activator of nuclear factor-κ B Ligand (RANKL, R&D) into the media, along with BSA-Au clusters that containing 10 μM, 50 μM and 100 μM of Au. The cells for differentiation induction were then incubated for 7 days. The indirect method was to pretreat MDA-MB-231 cells with BSA-Au clusters containing 10 μM, 50 μM and 100 μM of Au for 24 h before change the media into normal DMEM to eliminate the direct influences of BSA-Au clusters. After another 24 h, the supernatant fluids were collected and mixed with normal α-MEM at a 1:1 ratio for incubating with mouse BMMs for 48 h. The procedure was repeated four times for a total of 8 days osteoclastogenesis induction. The TRAP stain was conducted with a Leukocyte Acid Phosphatase kit (Sigma-Aldrich). The TRAP-positive cells were photographed and counted with an optical microscope (Olympus).

### Filamentous actin (F-actin) staining

Filamentous actin (F-actin) staining was conducted for the mouse BMMs that were treated with the direct method. The cells were washed twice with phosphate buffer saline (PBS, Gibco), then fixed in 3.7% paraformaldehyde (Aladdin) for 20 min and placed with 0.1% Triton X-100 (Molecular Devices) for 15 min at room temperature. The actin rings were stained with rhodamine-conjugated phalloidin (Cytoskeleton) and the cell nuclei were stained with Hoechst 33342 (Thermo Fisher Scientific) for 15 min. Actin ring formation in each sample was photographed and counted under a confocal laser scanning microscopy (Nikon Ti-E imaging system).

### Bone resorption assay

The pit formation assay was performed in Corning Osteo Assay Surface that mimic native bone *in vitro*. BMMs were seeded in 24-well plates coated with calcium phosphate substrate at a density of 2 × 10^4^ cells/well. After 24 h incubation, the medium was refreshed with medium containing different dose of BSA-Au clusters (10, 50 or 100 μM). The medium was then refreshed every two days. After about 7 days' incubation, cells were removed with 10% sodium hypochlorite and the plates were rinsed for three times with water. The bone resorption pits were observed and photographed by light microscope. The percentage of resorbed bone surface area was quantified by Image-Pro Plus 6.0 software.

### Colony formation assay

MDA-MB-231 cells were seeded in 12-well plates with 5×10^3^ cells per well, and incubated with 0, 10, 50, or 100 μM (dose of Au) of BSA-Au clusters for 14 days. Then, the cells were fixed for 0.5 h with 4% paraformaldehyde, stained with crystal violet (Solarbio) for 0.5 h at room temperature (25°C), washed two times with PBS buffer, and the numbers of colonies were photographed and counted under optical microscope (Olympus).

### RNA extraction and real-time PCR

Mouse BMMs treated with the direct method were collected for total RNA extraction with TRIzol reagent (Invitrogen). 500 ng total RNA of each group was reverse-transcribed with PrimeScript^TM^ RT Master Mix (Takara). The complementary DNA obtained was then subjected to real-time PCR with SYBR Premix Ex Taq (Takara). Primers used were listed in Table S1. A 2^-ΔΔCt^ method was performed for data analysis with normalization to the endogenous control β-actin.

### Protein extraction and western blotting analysis

The molecular mechanism of breast cancer bone metastasis is complex, involving the interaction between breast cancer cells and the bone microenvironment 37. Several vital osteomimetic factors produced by breast cancer cells within the bone microenvironment are responsible for bone metastasis and tumor-induced osteoclastogenesis, mainly including Osteoactvin, Cadherin-11, matrix metalloproteinase factor (MMP-9), C-X-C motif chemokine receptor type 4 (CXCR4), parathyroid hormone related protein (PTHrP), transforming growth factor-β (TGF-β) and runt-related transcription factor 2 (Runx2) 11,38. PTHrP can upregulated CXCR4 expression to drive migration and promote the secretion of RANKL to activate bone osteolysis 39,40. NF-κB signaling pathway is essential for metastatic breast cancer induced osteoclastogenesis in BMMs 41. In unstimulated cells, NF-κB is sequestered in the cytoplasm through interaction with inhibitory proteins IκB. Activated signals cause phosphorylation and subsequent degradation of IκB proteins, and the released NF-κB enters the nucleus to induce expression of specific target genes 42. Therefore, these proteins were detected in MDA-MB-231 cells and mouse BMMs by western blotting respectively. Total protein was extracted through RIPA Lysis Buffer (Beyotime). The protein samples were loaded equally in 10% sodium dodecyl sulfate gel prepared beforehand and been transferred to a PVDF membrane. The membrane was incubated with the targeted primary antibodies for 2 h at room temperature after being blocked with 5% BSA. Afterwards, it was incubated with horseradish peroxidase labeled secondary antibody (R&D) and scanned through LI-COR Infrared Imaged Odyssey.

### Transwell assay

Transwell assays were performed by using the Boyden chambers and Matrigel Matrix (Corning, 356234) following the instruction. Mouse BMMs or MDA-MB-231 cells were collected and re-suspended in blank medium after 12 h serum-free starvation. A total of 5×10^4^ cells were planted in the top chambers while the bottom chambers were filled with condition media and 2% of fetal bovine serum. Migrates cells were fixed with 3.7% paraformaldehyde (Aladdin) and stained with 0.1% crystal violet (Beyotime). Cells were then observed through an optical microscope (Olympus) and counted by Image-Pro Plus 6.0.

### Wound-healing assay

Wound-healing assays were performed by culturing 5×10^5^ MDA-MB-231 cells in each wells of 6-well plates. After incubating overnight, straight lines with a width of 0.5 cm were drawn at the bottom of the wells by pipette tips to erase the cells grew within. The cells were then incubated for another 12 h in DMEM with BSA-Au clusters containing 10 μM, 50 μM and 100 μM of Au before fixed with 3.7% paraformaldehyde (Aladdin) and stained with 0.1% crystal violet (Beyotime). Cells migrated into the erased area were observed through an optical microscope (Olympus) and counted by Image-Pro Plus 6.0.

### Allograft tumor model and treatment analysis

All animal care and experiments were approved by the Institutional Animal Care and Ethic Committee at the Chinese Academy of Sciences (Approved No. SYXK (Jing) 2018-0035). A total of 18 female BALB/c nude mice (2-3week, 15-17g) were purchased from Beijing Huafukang Bioscience CO. INC. The breast cancer bone metastasis model was established by direct injection of MDA-MB-231 cells (2×10^5^ cells in 20 μL PBS) into the right tibia of mice. The mice loaded with a tumor were then randomly divided into three groups (n = 6): saline group, 5 mg/kg BSA-Au clusters group, and 10 mg/kg BSA-Au clusters group. On the third day after injection of MDA-MB-231 cells, the mice were intraperitoneally administered of drugs every day for 20 days before been sacrificed. Body weights were measured every three days throughout the experiment. The right tibias were severed and first scanned by 3D micro computed tomography (microCT, Quantum GX, PerkinElmer, USA) at a voltage of 90 kV and an electric current of 88 µA and analyzed with AccuCT software (PerkinElmer, USA), then sliced up after been decalcified for pathologic analysis in histological sections. The tumor tissues were sliced up as well and the apoptosis of the cells in the tumor tissue was detected by TUNEL staining. All sections went through HE staining and immunohistochemical staining by indicated antibodies (Biovison). The expression of each protein in the histologic sections was quantized by Color Detection module of Immunohistochemistry (IHC) Image Analysis Toolbox in Image J software, according to the manufacturer's instructions 43. The histological sections were also performed TRAP staining by using the Leukocyte Acid Phosphatase kit (Sigma-Aldrich) and analyzed by Image-Pro Plus 6.0 software.

### Statistical analysis

All results in this series were recorded as mean ± standard deviation (SD). Significant differences between groups were determined by unpaired Student's t test with a p < 0.05 considered statistically significant. All analyses were carried out using the software SPSS for windows, version 23.0.0 (IBM corp).

## Results

### Characterization of BSA-Au clusters

**Figure 1:**
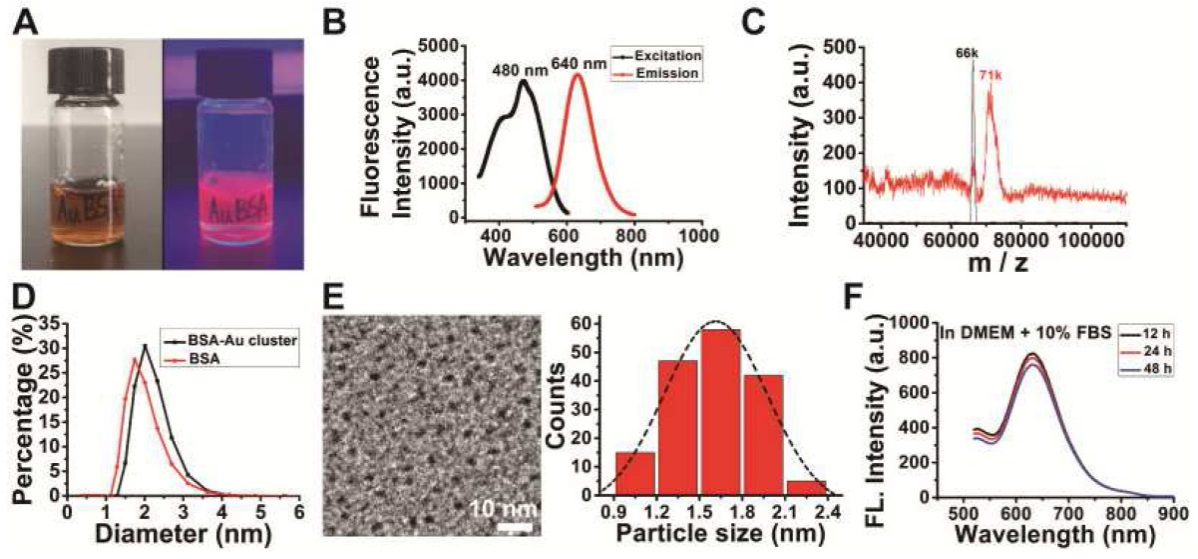
** Characterization of the synthesized BSA-Au clusters.** (A) The photographs of the BSA-Au clusters solution under visible light and UV light. (B) The fluorescence excitation and emission spectra of BSA-Au clusters (480 nm and 640 nm). (C) MALDI-TOF mass spectra of BSA (black) and BSA-Au clusters (red). (D) DLS spectra of the as-prepared BSA-Au clusters (black line) and BSA (red line). (E) The TEM image and core size distribution of the synthesized BSA-Au clusters. (F) Fluorescence emission of BSA-Au clusters at different time points in DMEM complete medium.

The purified BSA-Au clusters solution was a stable clear liquid substance at room temperature with the color of deep brown under daylight, and emitted an intense red fluorescence under 365 nm ultra violet light (Figure 1A). Fluorescence intensity detection shows the BSA-Au clusters has a fluorescence emission peak at the wavelength of 640 nm and an excitation peak at 480 nm (Figure 1B). MALDI-TOF mass spectrometry showed the molecular weight of as-prepared BSA-Au cluster to be ~71 kDa (Figure 1C), increased ~5 kDa from that of BSA (~66 kDa), which could be attributed to the 25 gold atoms in the Au cluster. The Au_25_ cluster was also indicated by the photoemission peak at 640 nm, based on the spherical Jellium model and previous reported 33,44. The hydrodynamic size of the prepared BSA-Au cluster was about 2.20 nm (Figure 1D, black line), slightly larger than that of BSA protein (~1.93 nm, red line). TEM image showed the core size of synthesized gold clusters is about 1.63 ± 0.31 nm (Figure 1E), which was consistent with that previously reported 34,35. There is no obvious change in fluorescence properties of BSA-Au cluster in various buffer solutions we used in this study over 48 h, including DMEM complete medium (Figure 1F), ultrapure water, saline and αMEM complete medium (Figure S1).

### BSA-Au clusters attenuate migration, invasion and colony formation of MDA-MB-231 cells *in vitro*

**Figure 2:**
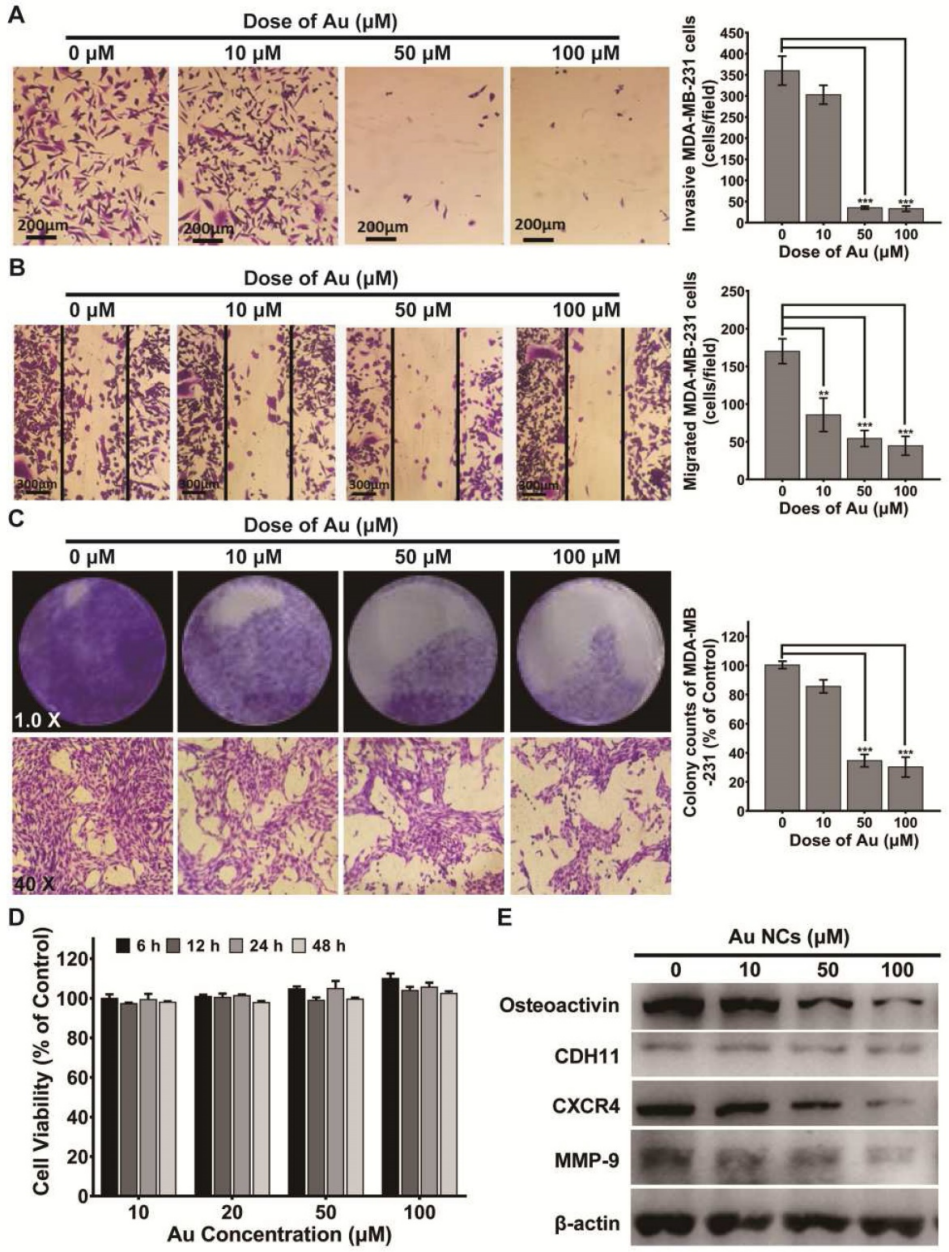
** Effect of BSA-Au clusters on the proliferation and metastasis process of MDA-MB-231 cells.** (A) Transwell assay of MDA-MB-231 cells and the quantitative analysis of Invasive cells, Data were from three independent experiments and one representative result is shown here (scale bar: 200 µm). The data are presented as mean ± standard deviation of triplicate experiments, **P < 0.01, ***P < 0.001 compared to the control group. (B) Wound healing assay of MDA-MB-231 cells and the quantitative analysis of migrated cells, Data were from three independent experiments and one representative result is shown here (scale bar: 300 µm). The data are presented as mean ± standard deviation of triplicate experiments, **P < 0.01, ***P < 0.001 compared to the control group. (C) Colony formation assay of MDA-MB-231 cells and the quantitative analysis of colony counts, Data were from three independent experiments and one representative result is shown here (up panel: Magnified 1.0 times; down panel: Magnified 40 times). The data are presented as mean ± standard deviation of triplicate experiments, ***P < 0.001 compared to the control group. (D) CCK-8 assay for cell viability of MDA-MB-231. The data is presented as mean ± standard deviation of triplicate experiments. (E) Western blotting detection of Osteoactivin, CDH11, CXCR4 and MMP-9 in MDA-MB-231 cells with or without treatment of BSA-Au clusters. β-actin was used as the loading control. Data were from three independent experiments and one representative result is shown here.

The capability of migration and invasion of tumor cells is crucial in the process of breast cancer bone metastases. So, we first explored if BSA-Au clusters would inhibit the MDA-MB-231cells' migration and invasion. The results of wound-healing assay and transwell assay showed that BSA-Au clusters significantly inhibited both migration and invasion of MDA-MB-231 cells *in vitro* at Au concentration of 50 μM or higher (Figure 2A-B). In addition to migration and invasion, the process of colonization is also crucial for the metastasis of breast cancer cells. We performed a colony formation experiment to determine the potential effects of the BSA-Au clusters on the colonization ability of MDA-MB-231 cells *in vitro*. Data revealed that treating with 50 μM or higher dose of BSA-Au clusters effectively inhibited the colony forming ability of MDA-MB-231 cells (Figure 2C). Next, the cytotoxicity of BSA-Au clusters to MDA-MB-231 cells was determined. CCK-8 assay indicated the viabilities of MDA-MB-231 cells were not obviously influenced by BSA-Au clusters within 48 h, at its Au concentration from 10 to 100 μM (Figure 2D), although the internalized Au did increase with the rise of BSA-Au clusters concentration detected by ICP-MS (Figure S2).

Several proteins overexpressed in bone-seeking breast cancer cells were associated with increased migration and invasiveness in bone metastasis, including Osteoactvin, Cadherin-11, MMP-9 and CXCR4. Hence, we evaluated the regulation of BSA-Au clusters on the expression of these proteins in MDA-MB-231 cells, which may explain the changes in tumor migration and invasive abilities. Results indicated that, after 48 h incubated with BSA-Au clusters, protein expressions of MMP-9, CXCR4 and Osteoactvin in MDA-MB-231 cells are significantly suppressed (Figure 2E).

### BSA-Au clusters inhibit tumor-induced mobilization and differentiation of osteoclast *in vitro*

**Figure 3:**
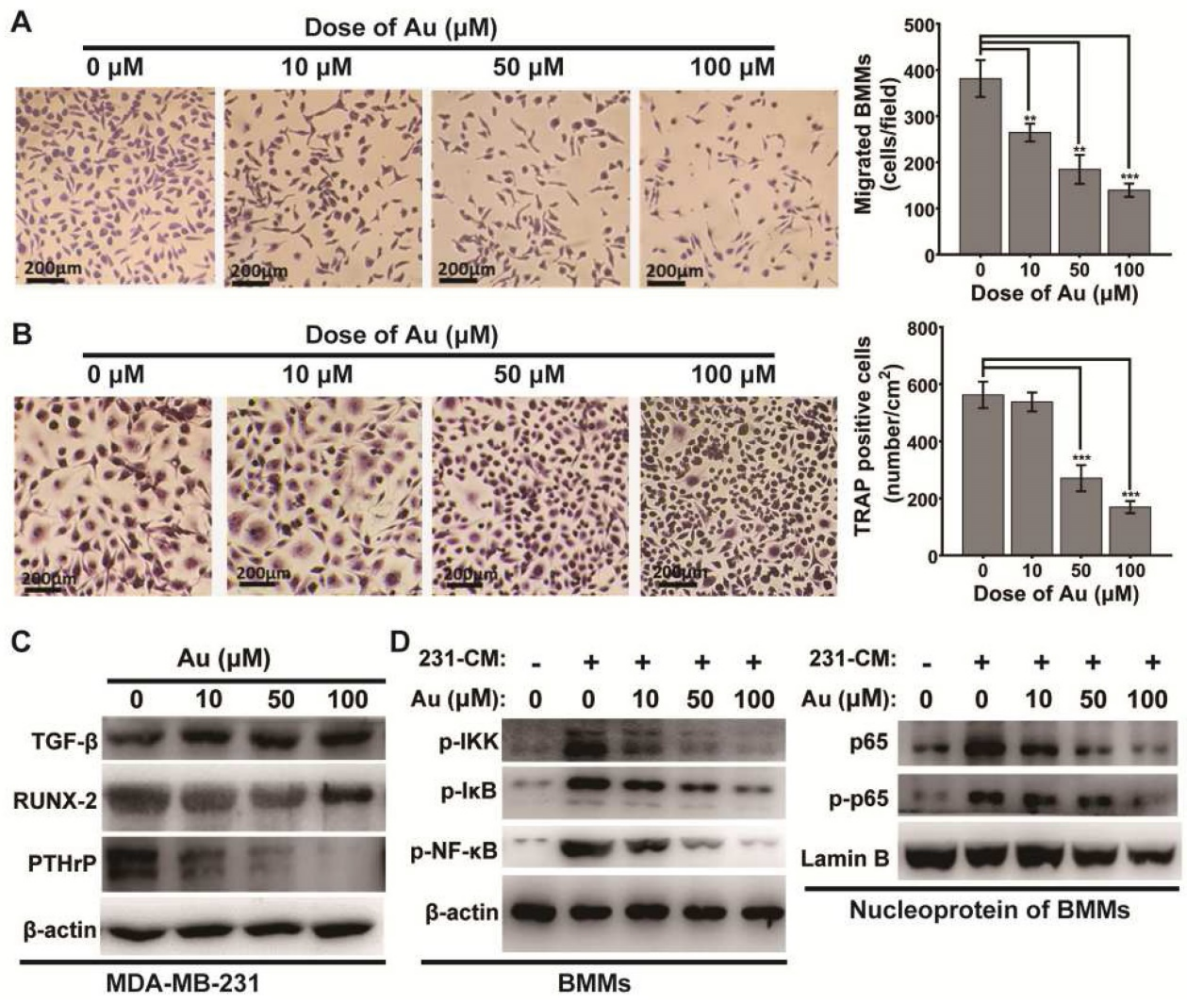
** Effect of BSA-Au clusters on MDA-MB-231 cells induced mobilization and differentiation of osteoclast *in vitro*.** (A) Tranwell assay of mouse BMMs induced by MDA-MB-231 supernatant and the quantitative analysis of migrated cells, Data were from three independent experiments and one representative result is shown here (scale bar: 200 µm). The data are presented as mean ± standard deviation of triplicate experiments, **P < 0.01, ***P < 0.001 compared to the control group. (B) TRAP stains of mouse BMMs derived osteoclasts induced by MDA-MB-231 supernatant and the quantitative analyses of TRAP^+^ cells, Data were from three independent experiments and one representative result is shown here (scale bar: 200 µm). The data are presented as mean ± standard deviation of triplicate experiments, ***P < 0.001 compared to the control group. (C) Western blotting detection of TGF-β RUNX-2 and PTHrP in MDA-MB-231 cells with or without treatment of BSA-Au clusters. β-actin was used as the loading control. Data were from three independent experiments and one representative result is shown here. (D) Western blotting detection of NF-κB pathway in MDA-MB-231 conditioned medium (231-CM) stimulated BMMs. Left panel: cytosol protein; right panel: nucleoprotein. β-actin and Lamin-B was used as the loading control. Data were from three independent experiments and one representative result is shown here.

Secondary osteolysis caused by breast cancer bone metastases is a vital step in the pathological progresses of the disease 37. This because tumor cells in metastatic bone site is able to secret cytokines that recruit bone marrow monocytes (BMMs) locally and induce osteoclastogenesis to form overloaded osteoclasts 45. To evaluate whether BSA-Au clusters could slow or block the pathological progress, we performed another transwell assay to assess the effects of BSA-Au clusters on MDA-MB-231 cells-induced recruiting of BMMs. Medium supernatant from MDA-MB-231 cells (MDA-MB-231 cell-conditioned medium) pretreated with or without BSA-Au clusters for 48 h was added to the bottom chambers to induce migration of BMMs. In order to eliminate the influence of the residual BSA-Au clusters in the supernatant on BMMs, after 48 h incubation with or without BSA-Au clusters, the culture medium of MDA-MB-231 cell was replaced by drug-free medium, and after 24 h incubation, the supernatant was transferred to the bottom chambers. Results indicated that the migrated mouse BMMs strongly decreased when the MDA-MB-231 cells pretreated with BSA-Au clusters, in a dose-dependent manner (Figure 3A).

Then, the effect of BSA-Au clusters on MDA-MB-231 cell-conditioned medium induced differentiation of osteoclast was evaluated by TRAP staining, after the BMMs were stimulated with the conditioned medium. Results indicated that the number of TRAP positives (TRAP^+^) cells was significantly decreased when MDA-MB-231 cells were pre-treated with BSA-Au clusters, in a dose-dependent manner (Figure 3B).

We then investigated how the BSA-Au clusters suppress MDA-MB-231 cell-induced osteoclastogenesis. We evaluated the regulation of BSA-Au clusters on expression of several vital osteomimetic factors produced by breast cancer cells in MDA-MB-231 cells. After 48 h incubated with BSA-Au clusters, expressions of PTHrP and Runx2, but not TGF-β, were significantly suppressed, in MDA-MB-231 cells (Figure 3C).

Once stimulated, the NF-κB signaling pathway in osteoclast precursor cells will be activated by MDA-MB-231 cell-conditioned medium, which regulates the differentiation of osteoclasts 46. We thus detected the influence of BSA-Au clusters on the activation of NF-κB signaling pathway in MDA-MB-231 cell-conditioned medium treated mouse BMMs by western blotting. The results indicated the MDA-MB-231 cell-conditioned medium induced phosphorylation of IKK, IκBα and p65, as well as the nuclear transposition of p65 were all suppressed by BSA-Au clusters treatment (Figure 3D).

### BSA-Au clusters inhibit RANKL-induced osteoclastic differentiation of mouse BMMs

RANKL, a member of the tumor necrosis factor (TNF) family, plays a central role in the development of osteoclasts and breast cancer bone metastasis 3. It is abundantly clear that metastatic breast cancer cells secreted inflammatory cytokines and PTHrP to upregulates RANKL expression in bone marrow stromal cells and osteoblasts, leading to increased osteoclastogenesis and enhanced pathological osteolysis 3,11. We further explored whether BSA-Au clusters could suppress the RANKL induced osteoclastogenesis in mouse BMMs.

**Figure 4:**
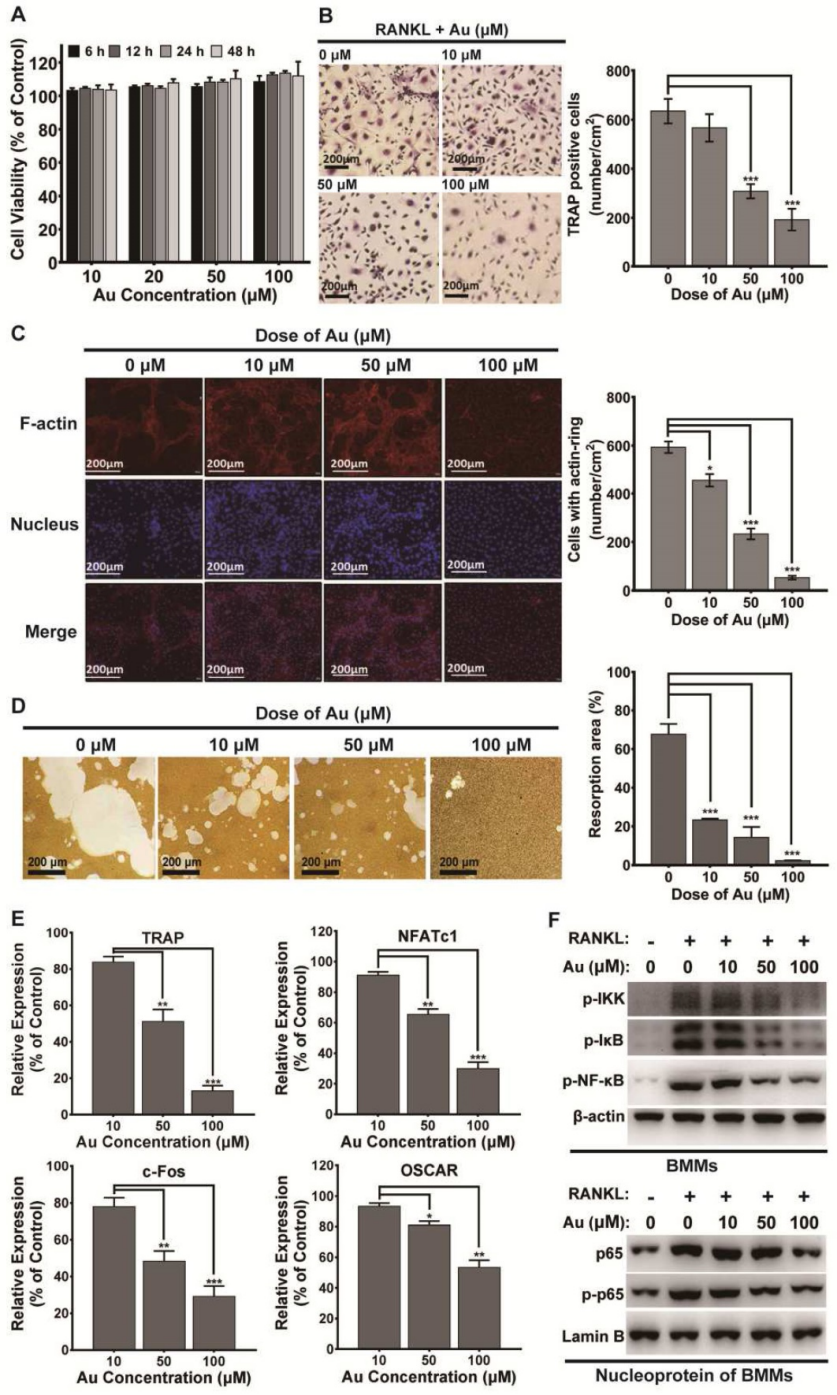
** Effect of BSA-Au clusters on RANKL-induced osteoclastogenesis of mouse BMMs *in vitro*.** (A) CCK-8 assay for cell viability of mouse BMMs. The data is presented as mean ± standard deviation of triplicate experiments. (B) TRAP stains of mouse BMMs derived osteoclasts induced by RANKL and the quantitative analyses of TRAP^+^ cells, Data were from three independent experiments and one representative result is shown here (scale bar: 200 µm). The data are presented as mean ± standard deviation of triplicate experiments, ***P < 0.001 compared to the control group. (C) F-actin staining of mouse BMMs derived osteoclasts induced by RANKL and the quantitative analyses of cells with actin ring, Data were from three independent experiments and one representative result is shown here (scale bar: 200 µm). The data are presented as mean ± standard deviation of triplicate experiments, *P < 0.05, ***P < 0.001 compared to the control group. (D) Representative images of bone resorption pits and the quantitative analysis of the ratio of resorption pits in unit area were presented. The data is presented as mean ± SD of triplicate experiments, *** P < 0.001. (E) The osteoclastogenesis marker genes TRAP, NFAT1, c-Fos and OSCAR were assessed by qRT-PCR, The data is presented as mean ± standard deviation of triplicate experiments, *P < 0.05, **P < 0.01, ***P < 0.001 compared to the control group. (F) RANKL-induced activation of NF-κB pathway in BMMs with or without treatment of BSA-Au clusters were assessed by western blotting. Up panel: cytosol protein; down panel: nucleoprotein. β-actin and Lamin-B was used as the loading control. Data were from three independent experiments and one representative result is shown here.

The potential cytotoxicity of BSA-Au clusters to mouse BMMs was first determined by CCK-8 assay. Data indicated the BSA-Au clusters had no obvious effect on cell viability of BMMs under RANKL stimulation (Figure 4A). Then, TRAP staining, F-actin staining and bone resorption assay *in vitro* was performed respectively. The results showed that the RANKL-induced osteoclastic differentiation of mouse BMMs was significantly inhibited by treating with BSA-Au clusters in a dose-dependent manner (Figure 4B-D). Both numbers of TRAP positive cells and F-actin positive cells decreased significantly after BSA-Au clusters treatments (Figure 4B-C).

Once activated, osteoclast differentiation is regulated by several genes, including TRAP, nuclear factor of activated T-cells cytoplasmic 1 (NFATc1), osteoclast associated receptor (OSCAR) and c-Fos. Thus, effects of BSA-Au clusters on RANKL-induced expression of these genes were detected by quantitative RT-PCR. Data revealed that the expressions of these osteoclast marker genes were increased by RANKL stimulation, but significantly attenuated by BSA-Au clusters treatments (Figure 4E).

NF-κB signaling pathway is the main early signal during RANKL-mediated osteoclastogenesis 47. Thus, RANKL-induced activation of intracellular NF-κB signaling pathway was examined, with or without treating with BSA-Au clusters. The results indicated that the RANKL-induced phosphorylation of IKK, IκBα and p65, as well as the nuclear transposition of p65 in mouse BMMs were all downregulated in the presence of BSA-Au clusters (Figure 4F).

### BSA-Au clusters prevent metastatic breast cancer induced osteolysis *in vivo*

**Figure 5:**
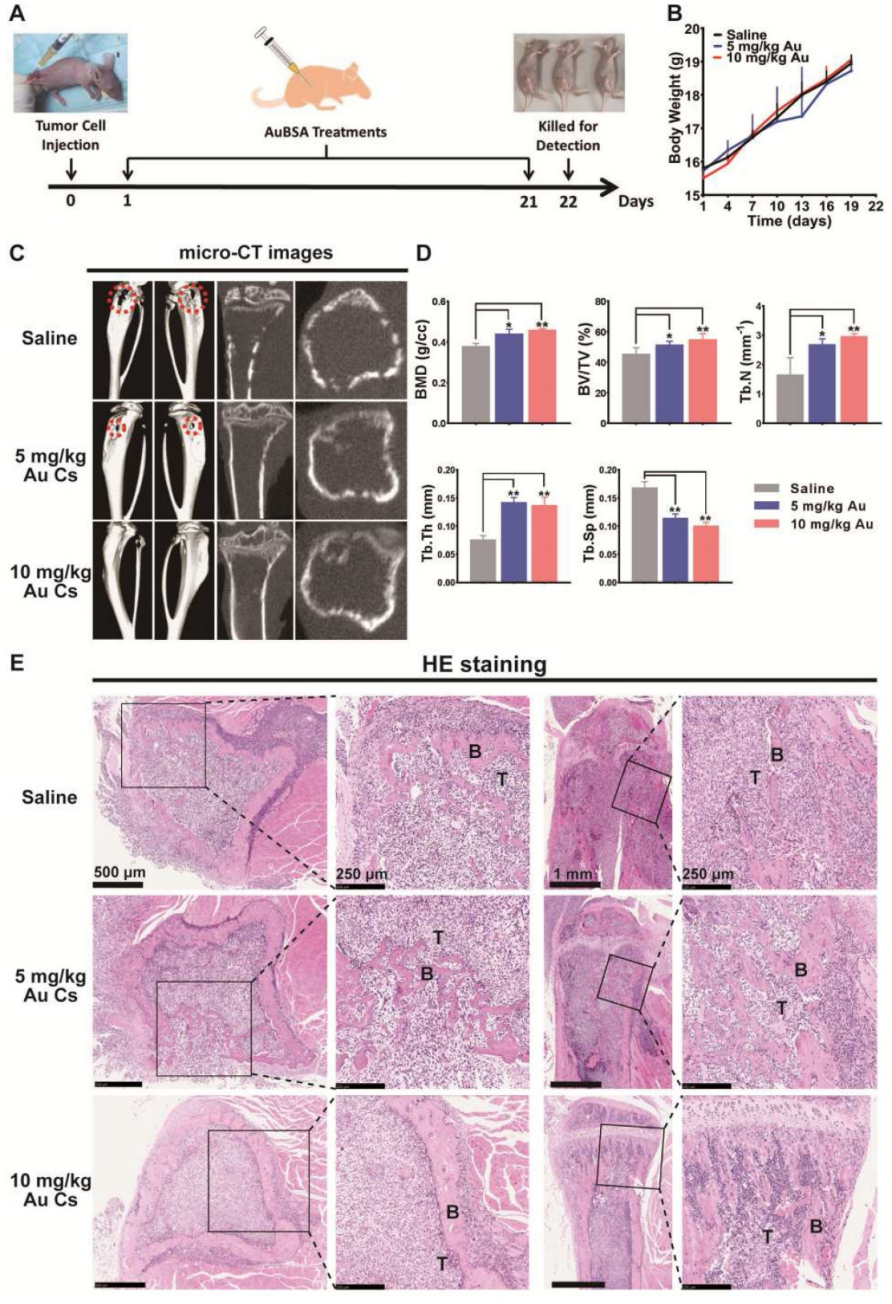
** Effects of BSA-Au clusters on metastatic breast cancer-induced osteolysis *in vivo*.** (A) Time axis of the *in vivo* treatment schedule. The breast cancer bone metastasis model in mouse was established by intramedullary injection of MDA-MB231 cells into the right hindlimb of nude mice. (B) Change of body weight in each group was measured every 3 days. The data is presented as mean ± Standard deviation. (C) Representative photographs of microCT observation in each group of mice treated with saline or BSA-Au clusters, n = 5 per group. The typical site of severe bone erosion is marked by red dotted cycle. (D) Bone histomorphometrics of proximal tibias in each group was quantitatively analyzed, the data are presented as mean ± Standard deviation, n = 5 per group, *P < 0.05, **P < 0.01. (E) Representative histopathological images of the tibia bone-tumor interface in each group treated with saline or BSA-Au clusters, n = 5 per group. T: tumor. B: bone.

In view of the dual inhibitory activities of BSA-Au clusters on tumor metastasis and osteoclast differentiation *in vitro*, we next evaluated the *in vivo* activity of BSA-Au clusters in a mouse model of breast cancer bone metastasis (Figure 5A). The bone metastasis model established by injecting breast cancer cells into the bone marrow cavity of the tibia is well applied to study the interactions between cancer cells and the bone microenvironment 14. Visible tumor lesions that can be observed were grown around the right tibia of mice at approximately 1 week after the injection. And the mice were then showing symptoms such as lameness and lassitude at varied degrees. We detected no significant changes in body weight in mice injected with BSA-Au clusters and the control group during the whole experiment (Figure 5B). As showed by the micro-CT observation, the tumor induced obvious bone erosion on the surfaces and inside of the tumor-loaded tibias in mice of the untreated group (Figure 5C). Treating with 10 mg/kg BSA-Au clusters significantly relieved the osteolysis on the surfaces and inside of the tibias, although the dose of 5 mg/kg showed slighter improvement (Figure 5C). Bone histomorphometrics of each group was analyzed, including bone mineral density (BMD), bone volume/tissue volume ratio (BV/TV), trabecular number (Tb.N.), trabecular thickness (Tb.Th.) and trabecular separation (Tb.Sp). Data suggested that the bone metrics in the groups treated with BSA-Au clusters were significantly higher than in the groups treated with saline, and 10 mg/kg BSA-Au clusters showed better therapeutic effect (Figure 5D). We then performed a HE staining in histological sections to observe the tumor-bone interface and found that the tumor-induced osteolysis in BSA-Au clusters treated groups were less aggressive than that in the saline treated group (Figure 5E). However, treating with BSA-Au clusters did not obviously influence the growth or apoptosis of tumor cells *in vivo*, which was consistent with the *in vitro* cytotoxicity evaluation in cultured MDA-MB-231 cells (Figure S3).

**Figure 6:**
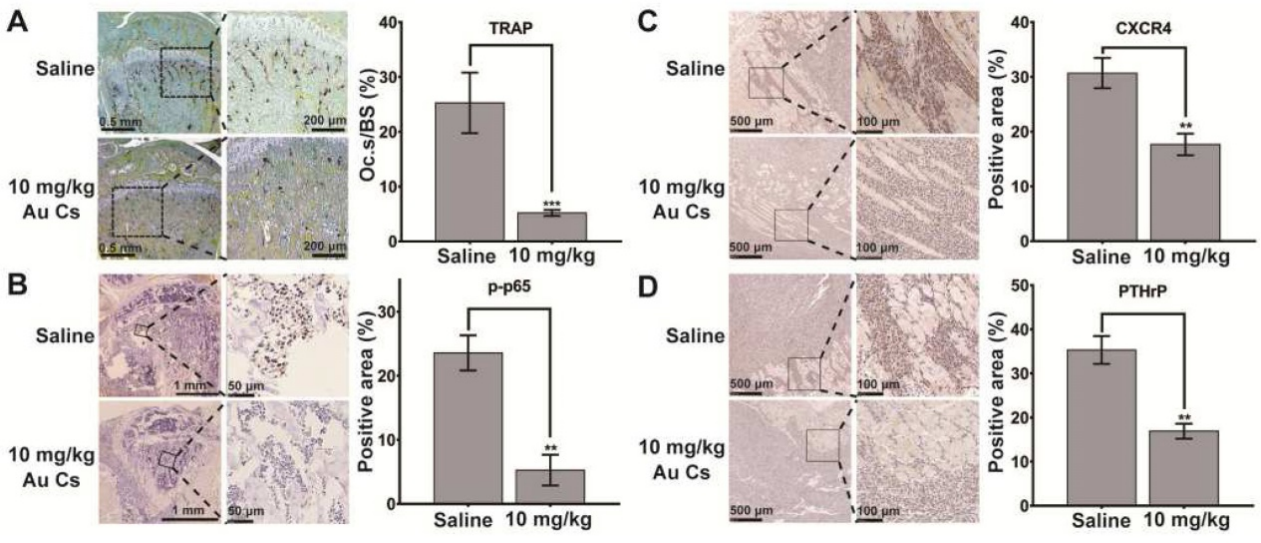
** Effects of BSA-Au clusters on the tumor-induced osteoclastogenesis and expression of marker proteins *in vivo*.** Immunohistochemical staining of the bone-tumor interface within the tibia in saline and 10 mg/kg BSA-Au clusters treated group. (A) Representative histological view of MDA-MB-231 cell induced osteoclastogenesis within tibia and the quantitative analyses of relative positive area. TRAP-positive (stained purple) multinucleated osteoclasts are lining along the bone surface. Osteoclast surface/bone surface (Oc.S/BS) were quantitative analyzed and the data is presented as mean ± standard deviation, ***P < 0.001 compared to the saline group, n=3. (B) Immunohistochemical staining of phosphorylated-p65 (p-p65) and the quantitative analyses of relative positive area, one representative photo is shown here, n = 3. The data is presented as mean ± standard deviation, **P < 0.01 compared to the saline group. (C) Immunohistochemical staining of CXCR4 and the quantitative analyses of relative positive area, one representative photo is shown here, n = 3. The data is presented as mean ± standard deviation, **P < 0.01 compared to the saline group. (D) Immunohistochemical staining of PTHrP and the quantitative analyses of relative positive area, one representative photo is shown here, n = 3. The data is presented as mean ± standard deviation, **P < 0.01 compared to the saline group.

To elucidate the possible mechanism of action *in vivo*, according to the analysis in cultured cells, immunohistochemistry staining of PTHrP and CXCR4 in tumor cells and p-p65 in bone cells as well as TRAP staining were performed in the histological sections of tumor-loaded tibiae. The results indicated that the tumor-induced osteoclastogenesis and activation of p65 in bone tissues as well as the expression of PTHrP and CXCR4 in tumor cells were all suppressed after BSA-Au clusters treatment, which was consistent with the results detected *in vitro* (Figure 6A-D).

## Discussion

Cancer bone metastasis is the overwhelming cause of death in breast cancer patients 1. Serious skeletal-related events (SREs) caused by bone metastasis of breast cancer have a decisive impact on patients' morbidity and mortality 6. The occurrence of breast cancer bone metastasis is a complex process, involving the crosstalk between the disseminated cancer cells and bone cells 9,10. Therefore, in the process of treatment, it is very important to explore new chemical agents to reduce the metastasis of cancer cells and prevent the deterioration of bone.

Biomolecule-protected gold cluster is a novel gold nanomaterial with ultrasmall size (smaller than 2 nm) and well-defined molecular structure, making them possess various unique physicochemical and biomedical properties that are not seen in the corresponding bigger nanomaterials or bulk materials 18. In previous studies, we found that peptide-coated gold clusters possessed anti-osteoclastogenesis activity and prevented inflammation induced bone destruction effectively *in vivo* and revealed potential anti-tumor activities in certain cancer cells 23-29. Considering the key role of tumor-induced osteoclastogenesis in bone metastasis of breast cancer, we speculate the gold clusters may provide an exciting strategy to treat breast cancer bone metastasis. Albumin is widely used in applications of nano-pharmaceutics for its excellent biocompatibility and albumin-bound paclitaxel (Abraxane) has been approved to enter the clinical application 30,32. Therefore, a bovine serum albumin (BSA) coated gold cluster (BSA-Au clusters) was prepared in this study to evaluate its therapeutic activity in metastatic breast cancer induced osteoclastogenesis and osteolysis. We proved that the BSA-Au clusters could strongly inhibit migration, invasion and colonization of breast cancer cells as well as tumor-induced osteoclastogenesis *in vitro* by suppressing the expression of these osteomimicry factors.

Breast cancer cells express key osteogenic factors, which can deregulate the recruitment, differentiation and function of osteoclasts, and promote the homing, invasion, colonization, survival and proliferation of breast cancer cells within bone 37,38. The factors include Osteoactivin, MMP-9, CXCR4, Runx2 and PTHrP. Osteoactivin is associated with increased mobility, invasiveness and osteolytic bone metastasis formation *in vivo* 48. The expression of MMP-9 is related to the colonization of tumor cells in bone site and the occurrence of osteolytic lesions 49. CXCR4 mediates cancer cells metastasis to bone and plays a causal role in the formation of osteolytic lesions 50. Runx2 and PTHrP are usually highly expressive in metastatic breast cancer cells and act as promoters in tumor-induced osteoclastogenesis 40,51. Signaling protein PTHrP secreted by breast cancer cells promotes the secretion of RANKL to activate osteoclastic osteolysis 37. In most metastatic bone tumor lesions, the differentiation of osteoclast from BMMs are mainly mediated by activation of NF-κB pathway, which initiated by factors secreted in bone microenvironment by cancer cells and osteoblasts 41. In this study, we found that the as-prepared BSA-Au cluster could effectively inhibit the NF-κB pathway activated by RANKL or supernatant of cultured MDA-MB-231 cells in BMMs, which may responsible for the inhibition function on osteoclastogenesis. On the other hand, NF-κB pathway is also essential for metastasis progression in breast cancer cells 52,53. The suppression effects of gold cluster on metastasis of breast cancer cells *in vitro* maybe also due to the inhibition of intracellular NF-κB pathway, at least in part. The cellular viability of BMMs was not affected by BSA-Au clusters at these doses tested, indicated it is an agent that can target and block osteoclastogenesis without paying the cost of BMMs' cellular apoptosis. In previous studies, we have demonstrated another two peptide-coated gold clusters (GSH-Au cluster and Sv-Au cluster) also suppress the activation of NF-κB pathway in macrophage or BMMs 23, 24. These results suggested the biomedical activity of gold clusters mainly depends on their intrinsic biochemical properties, rather than the biomolecules and size.

An ideal model for breast cancer bone metastasis should reproduce all the stages of tumorigenesis and bone metastasis. To date, no mice model can accurately recapitulates breast cancer bone metastasis that happened in clinic 14. The xenograft models by injecting human cancer cells into immune-deficient mice are the present paradigms for modeling bone metastasis in mouse. These xenograft models can be categorized to orthotopic, intraosseous, intracardiac, intravenous, or subcutaneous, based on the injection site 14. Mammary orthotopic injection model is most anatomically similar to the process happened in clinic. However, bone metastases are rarely occurred in these orthotopic mouse models may due to the different between the microenvironments of human and the mouse 14. Intracardiac, intravenous, or subcutaneous injections can mimic spontaneous bone metastasis better than intraosseous injections. However, metastases most commonly form in the lungs in these models, because of the tumor cells are direct injected into the blood stream and lung metastasis can be achieved without the steps of invasion and intravasation 54. Compared with the above models, intraosseous injections are easier to induce tumor growth in the bone 13. This model is well established to study the interactions between breast cancer cells and the bone microenvironment. In this study, we focused on the potential intervention effects of gold cluster on the interactions between breast cells and osteoclast within the bone microenvironment, based on the results of *in vitro* co-culture assays. Therefore, intraosseous injection model was chose in this study. In this model, mice treated with BSA-Au clusters had experienced less aggressive bone erosion and did not reveal obvious loss of body weight during the whole treatment course. Cancer secreted PTHrP and CXCR4 and the activation of NF-κB as well as tumor-induced osteoclastogenesis within bone tissue were suppressed in the pathological samples after BSA-Au clusters treatments.

This study revealed that the BSA-Au cluster can inhibit both breast cancer cell metastasis and tumor-induced osteoclast differentiation and osteolysis, which are not available in recent therapeutic drugs, such as bisphosphonates have well-defined antiresorptive activity, but have little effect on tumor cells. There are several merits of the BSA-Au cluster can facilitate its clinical application in the future. First, the gold cluster is prepared by a facile one-pot “green” synthetic route with a common commercially available protein, making it suitable for large-scale stable preparation and considerable environmental/cost advantage 33,55. Second, the BSA coating layer can facilitate post-synthesis surface modifications with functional ligands to improve its targeting or therapeutic efficiency. Third, the BSA-Au cluster has high stability in both solution and solid state. Previous study has demonstrated the BSA-Au cluster could be stored in solid form for at least 2 months and re-dispersed when needed 33. This high stability would greatly facilitate its use in clinical applications. Last but important, good biocompatibility of the protein-protected Au clusters can promote their clinical transformation 18,35. Considering the potential application in clinic, the BSA can be replaced by the human serum albumin (HSA), which is highly homologous with BSA and has been successfully used in clinical medicine (Abraxane) 30. However, BSA-Au clusters did not reveal growth inhibiting activity on breast cancer cells, either *in vitro* or *in vivo*. In the future research, the cytotoxicity to metastatic breast cancer cells of gold clusters may be improved by optimizing the ligands and synthesis conditions.

## Conclusion

In summary, a BSA-coated gold cluster was synthesized to evaluate its therapeutic activity in metastatic breast cancer induced osteoclastogenesis *in vitro* and osteolysis* in vivo*. The BSA-Au clusters were able to inhibit breast cancer cell-induced and RANKL-induced osteoclast differentiation as well as the migration and invasion of breast cancer cells *in vitro*. Moreover, this gold cluster could relieve cancer induced osteolytic bone resorption in a mouse model of breast cancer bone metastasis by improving the bone microenvironment. Based on these results, gold clusters may provide a promising therapeutic strategy for treating bone metastasis of breast cancer.
