# Supplementary material for: Gold clusters prevent breast cancer bone metastasis by suppressing tumor-induced osteoclastogenesis
Source: Theranostics. 2020 Mar 4;10(9):4042–55. doi: 10.7150/thno.42218 (PMC7086366; doi:10.7150/thno.42218)
Supplement: Supplementary file 1 — Supplementary figures and table. [file thnov10p4042s1.pdf]

Supplementary data

Table S1. The primer sequences used in the study

Target Gene	Primer sequences (5'-3')
β-actin-F	GTACGCCAACACAGTGCTG
β-actin-R	CGTCATACTCCTGCTTGCTG
Mouse TRAP-F	GCTGGAAACCATGATCACCT
Mouse TRAP-R	GAGTTGCCACACAGCATCAC
Mouse NFATc1-F	TGGAGAAGCAGAGCACAGAC
Mouse NFATc1-R	GCGGAAAGGTGGTATCTCAA
Mouse OSCAR-F	CTGCTGGTAACGGATCAGCTCCCCAGA
Mouse OSCAR-R	CCAAGGAGCCAGAACCTTCGAAACT
Mouse C-FOS-F	TTGCTGAGTCGCTTGACTGG
Mouse C-FOS-R	GGATTTGACTGGAGGTCTGC

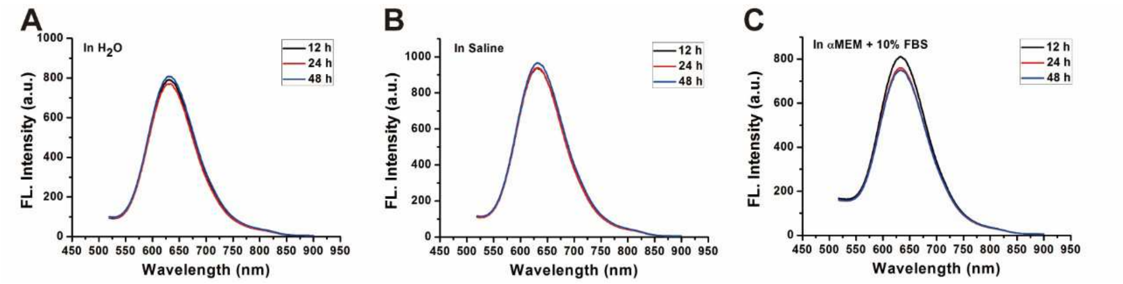

Figure S1. **Fluorescence emission of BSA-Au clusters at different time points in different buffer solutions**: ultrapure water (A), saline (B) and αMEM complete medium (C).

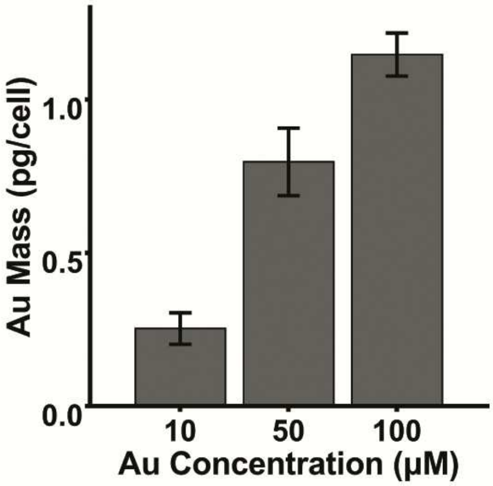

Figure S2. ICP-MS detection for intracellular Au content in MDA-MB-231 cells treated with different dose of BSA-Au clusters. The data is presented as mean ± standard deviation of triplicate experiments.

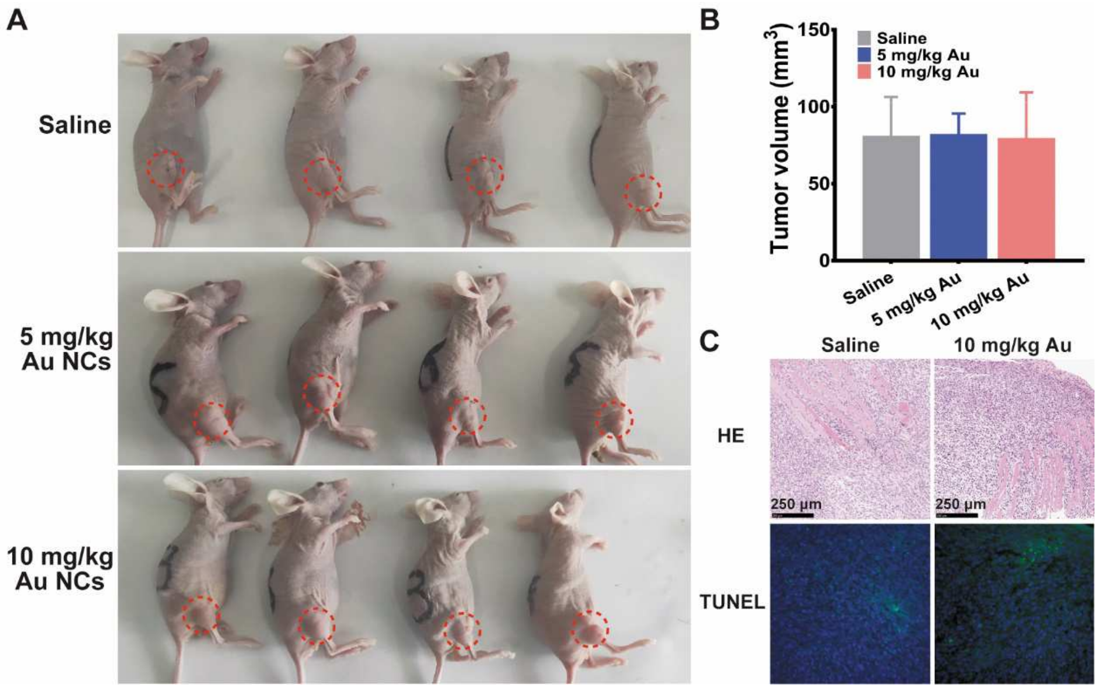

Figure S3. Tumor growth in each group of BALB/c nude mice. (A) Photographs of tumor-loading mice in each group after execution. Tumor site on the hindlimb is marked by red dotted cycle. (B) Quantitative analyses of tumor volume. The data is presented as mean ± standard deviation, n = 5. (C) HE and TUNEL staining of the tumor tissue of mice in the saline and 10 mg/kg BSA-Au clusters treated group.
